# A Review of Feature Selection Methods for Machine Learning-Based Disease Risk Prediction

**DOI:** 10.3389/fbinf.2022.927312

**Published:** 2022-06-27

**Authors:** Nicholas Pudjihartono, Tayaza Fadason, Andreas W. Kempa-Liehr, Justin M. O'Sullivan

**Affiliations:** ^1^ Liggins Institute, University of Auckland, Auckland, New Zealand; ^2^ Maurice Wilkins Centre for Molecular Biodiscovery, Auckland, New Zealand; ^3^ Department of Engineering Science, The University of Auckland, Auckland, New Zealand; ^4^ MRC Lifecourse Epidemiology Unit, University of Southampton, Southampton, United Kingdom; ^5^ Singapore Institute for Clinical Sciences, Agency for Science, Technology and Research (A*STAR), Singapore, Singapore; ^6^ Australian Parkinson’s Mission, Garvan Institute of Medical Research, Sydney, NSW, Australia

**Keywords:** machine learing, feature selection (FS), risk prediction, disease risk prediction, statistical approaches

## Abstract

Machine learning has shown utility in detecting patterns within large, unstructured, and complex datasets. One of the promising applications of machine learning is in precision medicine, where disease risk is predicted using patient genetic data. However, creating an accurate prediction model based on genotype data remains challenging due to the so-called “curse of dimensionality” (i.e., extensively larger number of features compared to the number of samples). Therefore, the generalizability of machine learning models benefits from feature selection, which aims to extract only the most “informative” features and remove noisy “non-informative,” irrelevant and redundant features. In this article, we provide a general overview of the different feature selection methods, their advantages, disadvantages, and use cases, focusing on the detection of relevant features (i.e., SNPs) for disease risk prediction.

## 1 Introduction

### 1.1 Precision Medicine and Complex Disease Risk Prediction

The advancement of genetic sequencing technology over the last decade has re-ignited interest in precision medicine and the goal of providing healthcare based on a patient’s individual genetic features (Spiegel and Hawkins, 2012). Prediction of complex disease risk (e.g., type 2 diabetes, obesity, cardiovascular diseases, etc…) is emerging as an early success story. Successful prediction of individual disease risk has the potential to aid in disease prevention, screening, and early treatment for high-risk individuals (Wray et al., 2007; Ashley et al., 2010; Manolio, 2013).

Genome-wide association studies (GWAS) have identified single nucleotide polymorphisms (SNPs) within the human genome that are associated with complex diseases at the population level (Altshuler et al., 2008; Donnelly, 2008; Hindorff et al., 2009). However, most of the SNPs that have been associated with phenotypes have small effect sizes (Visscher et al., 2017), and collectively they only explain a fraction of the estimated heritability for each phenotype (Makowsky et al., 2011). This is known as the *missing heritability* problem. One possible explanation for the missing heritability is that GWAS typically utilize univariate filter techniques (such as the χ^2^ test) to evaluate a SNP’s association with a phenotype SNP separately (Han et al., 2012). While univariate filter techniques are popular because of their simplicity and scalability, they do not account for the complex interactions between SNPs (i.e., epistasis effects). Ignoring interactions amongst genetic features might explain a significant portion of the missing heritability of complex diseases (Maher, 2008; König et al., 2016). Furthermore, being population-based, GWAS do not provide a model for predicting individual genetic risk. Thus, translation of GWAS association to individualized risk prediction requires quantification of the predictive utility of the SNPs that are identified. Typically, genetic risk prediction models are built by: 1) Polygenic risk scoring; or 2) Machine learning (ML) (Abraham and Inouye, 2015).

### 1.2 Machine Learning for Individualized Complex Disease Risk Prediction

**FIGURE 1:**
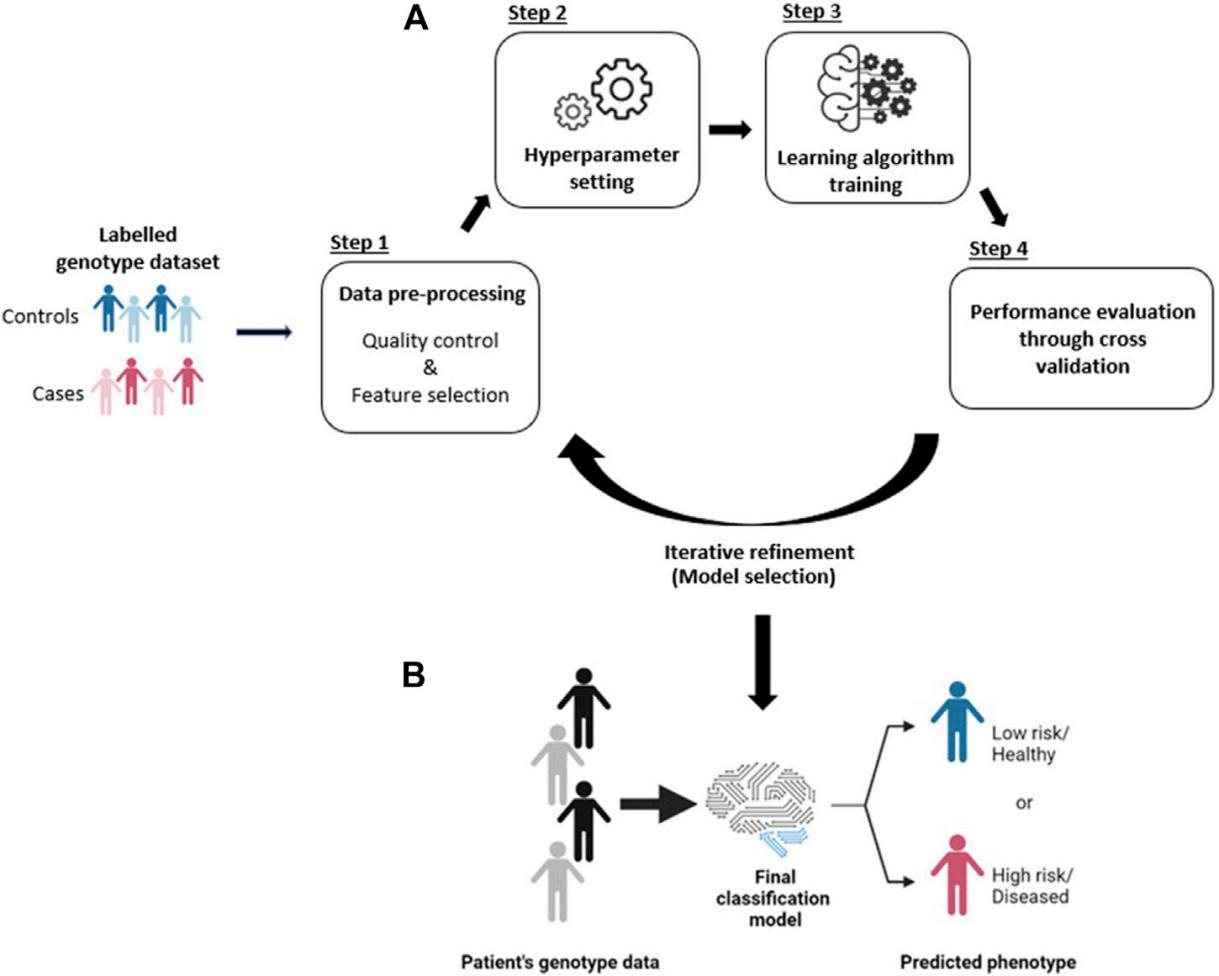
**(A)** Generalized workflow for creating a predictive ML model from a genotype dataset. **(B)** The final model can then be used for disease risk prediction.

ML-based approaches are a potentially effective way of predicting individualized disease risk (Figure 1). Unlike other popular predictive models (e.g., Polygenic Risk Scores, which use a fixed additive model), ML has the potential to account for complex interactions between features (i.e. SNP-SNP interaction) (Ho et al., 2019). ML algorithms utilize a set of advanced function-approximation algorithms (e.g., support-vector machine, random forests, K-nearest neighbor, artificial neural network, etc…) to create a model that maps the association between a set of risk SNPs and a particular phenotype (Kruppa et al., 2012; Mohri et al., 2018; Uddin et al., 2019). Thus, a patient’s genotype data can be used as an input to the predictive ML algorithm to predict their risk for developing a disease (Figure 1B).

The prediction of disease risk using SNP genotype data can be considered as a binary classification problem within supervised learning. There is a generalized workflow for creating a predictive ML model from a case-control genotype dataset (Figure 1A). The first step is data pre-processing, which includes quality control and feature selection (Figure 1A, step 1). Quality control includes, but is not limited to, removing low-quality SNPs (e.g., those with low call rates or that deviate from the Hardy-Weinberg Equilibrium), and samples (e.g. individuals with missing genotypes). SNPs with low minimum allele frequency (e.g., less than 0.01) can also be removed. Feature selection reduces the training dataset’s dimensionality by choosing only features that are relevant to the phenotype. Feature selection is crucial in order to produce a model that generalizes well to unseen cohorts (see Section 1.3). The goal of data pre-processing is to produce a high-quality dataset with which to train the prediction model.

The second step in a generalized predictive ML modelling workflow is the selection of the specific learning algorithm and setting the learning parameters (i.e. the “hyperparameters”) (Figure 1A, step 2). Hyperparameters are algorithm-specific parameters whose values are set before training. Examples include the number of trees in a random forest, the type of kernel in an SVM, or the number of hidden layers in an artificial neural network. Different learning algorithms use different hyperparameters, and their values affect the complexity and learning behaviour of the model.

Once the hyperparameters have been set, the pre-processed dataset is used to train the chosen algorithm (Figure 1A, step 3). This training step allows the algorithm to “learn” the association between the features (i.e., SNPs) and the class labels (i.e., phenotype status). Once learnt, the trained model’s predictive performance (e.g. accuracy, precision, AUC) is validated (Figure 1A, step 4). This is typically performed by K-fold cross-validation to estimate the model’s performance on unseen data. Cross-validation on unseen data ensures that the trained model does not overfit the training data. During cross-validation, the training dataset is equally split into K parts, and each part will be used as a validation/testing set. For example, in 5-fold (K = 5) cross-validation, the dataset is divided into 5 equal parts. The model is then trained on four of these parts and the performance is tested on the one remaining part. This process is repeated five times until all sections have been used as the testing set. The average performance of the model across all testing sets is then calculated.

The estimated model performance from cross-validation can be used as a guide for iterative refinement. During iterative refinement different aspects of the model building process (step 1–4) are repeated and refined. For example, different: hyperparameters (hyperparameter tuning); learning algorithms, feature selection methods, or quality control thresholds can all be tried. The combination that produces the best average performance (in cross-validation) is chosen to build the final classification model. The process of selecting the best model development pipeline is known as model selection. The final classification model can then be tested against an independent (external) dataset to confirm the model’s predictive performance, and finally be used for disease risk prediction (Figure 1B).

### 1.3 Feature Selection to Reduce SNP Data Dimensionality

Overcoming the curse of dimensionality is one of the biggest challenges in building an accurate predictive ML model from high dimensional data (e.g. genotype or GWAS data). For example, a typical case-control genotype dataset used in a GWAS can contain up to a million SNPs and only a few thousands of samples (Szymczak et al., 2009). Using such data directly to train the ML classification algorithms is likely to generate an overfitted model, which performs well on the training data but poorly on unseen data. Overfitting happens when the model picks up the noise and random fluctuations in the training data as a learned concept. Furthermore, the excessive number of features increases the learning and computational time significantly because the irrelevant and redundant features clutter the learning algorithm (Yu and Liu, 2004).

**FIGURE 2:**
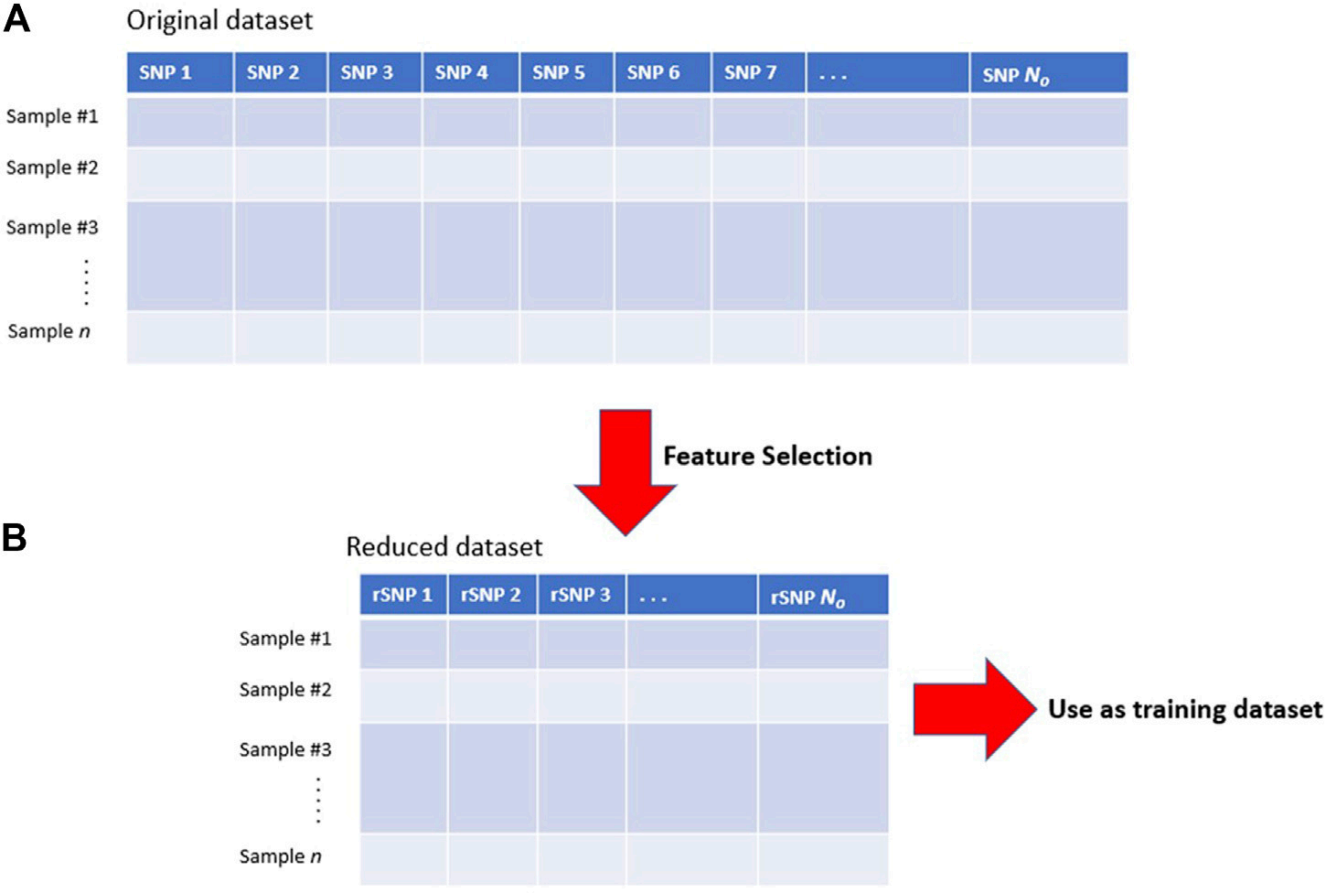
Illustration of feature selection process. **(A)** The original dataset may contain an excessive number of features and a lot of irrelevant SNPs. **(B)** Feature selection reduces the dimensionality of the dataset by excluding irrelevant features and including only those features that are relevant for prediction. The reduced dataset contains relevant SNPs (rSNPs) which can be used to train the learning algorithm. N_o_: original number of features, N_r_: number of remaining relevant SNPs.

Feature selection is a common way to minimize the problem of excessive and irrelevant features (Figure 2). Generally, feature selection methods reduce the dimensionality of the training data by excluding SNPs that: 1) have low or negligible predictive power for the phenotype class; and 2) are redundant to each other (Okser et al., 2014). Effective feature selection can increase learning efficiency, predictive accuracy, and reduce the complexity of the learned results (Koller and Sahami, 1996; Kohavi and John, 1997; Hall, 2000). Furthermore, the SNPs that are incorporated into the predictive model (following feature selection) are typically assumed to be associated with loci that are mechanistically or functionally related to the underlying disease etiology (Pal and Foody, 2010; López et al., 2018). Therefore, extracting a subset of the most relevant features (through feature selection) could help researchers to understand the biological process(es) that underlie the disease (Cueto-López et al., 2019). In this context, feature selection can be said to be analogous to the identification of SNPs that are associated with phenotypes in GWAS.

### 1.4 The Problem of Feature Redundancy and Feature Interaction in SNP Genotype Dataset

**FIGURE 3:**
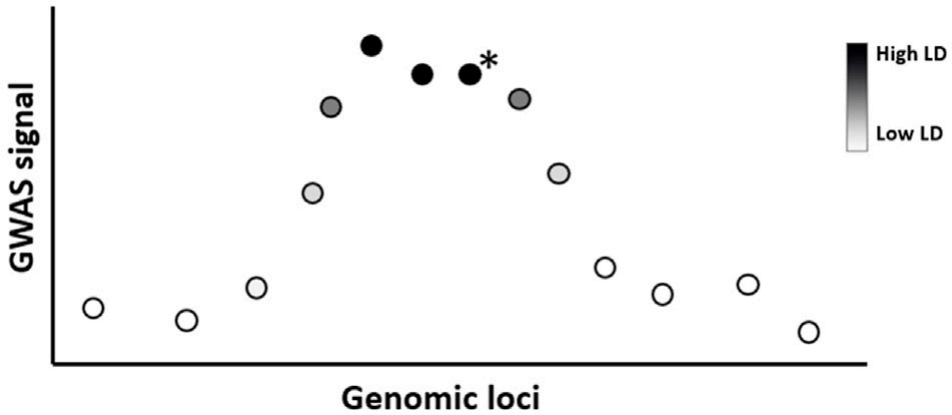
Lead SNPs in GWAS studies need not be the causal variant due to linkage disequilibrium. Illustration of GWAS result where SNPs (circles) are colored according to linkage disequilibrium (LD) strength with the true causal variant within the locus (indicated with a black star). Due to LD, several SNPs near the true causal variant may show a statistically significant association with the phenotype. In ML, these highly correlated SNPs can be considered redundant to each other, therefore only one representative SNP for this LD cluster is required as a selected feature. In this example, the causal variant is not the variant with the strongest GWAS association signal.

GWAS typically identify multiple SNPs close to each other within a genetic window to be associated with a disease (Broekema et al., 2020). This occurs because of linkage disequilibrium (LD), which is the correlation between nearby variants such that they are inherited together within a population more often than by random chance (Figure 3). In ML and prediction contexts, these highly correlated SNPs can be considered redundant because they carry similar information and can substitute for each other. The inclusion of redundant features has been shown to degrade ML performance and increase computation time (Kubus, 2019; Danasingh et al., 2020). Therefore, ideally, feature selection techniques should select one SNP (e.g., the SNP with the highest association score) to represent the entire LD cluster as a feature for prediction. However, since the SNP with the highest association signal is not necessarily the causal variant of that locus (Onengut-Gumuscu et al., 2015), geneticists often link an association signal to the locus they belong to rather than the SNP itself (Brzyski et al., 2017). If a researcher aims to identify the true causal variant within an association locus then fine-mapping techniques must be employed (see (Spain and Barrett, 2015; Broekema et al., 2020))

**FIGURE 4:**
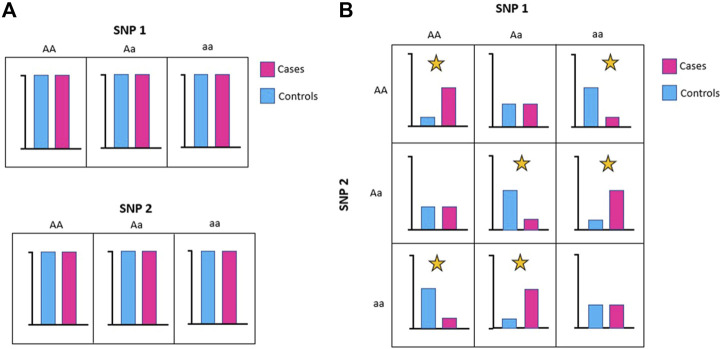
The functional impacts of SNPs can interact and may be epistatic. **(A)** Individually, neither SNP1 nor SNP2 affect phenotype distribution. **(B)** Taken together, allele combinations between SNP1 and SNP2 can affect phenotype distribution (marked with yellow star).

Relevant features may appear irrelevant (or weakly relevant) on their own but are highly correlated to the class in the presence of other features. This situation arises because these features are only relevant to the phenotype when they interact with other features (i.e., they are epistatic). Figure 4 shows a simplified example of a feature interaction that arises because of epistasis. In this example, there is an equal number of SNP 1 = AA, Aa, or aa in cases and controls, which means that SNP 1 does not affect the distribution of the phenotype class. The same is true for SNP 2. However, the allele combinations between SNP1 and SNP2 does affect phenotype distribution. For example, there are more combinations of SNP1 = AA and SNP2 = AA in cases than controls, consistent with this allele combination conferring increased risk (Figure 4B).

It is generally advisable to consider both feature redundancy and feature interaction during feature selection. This is especially true when dealing with genotype data, where linkage disequilibrium (LD) and the non-random association of alleles create redundant SNPs within loci. Moreover, complex epistatic interactions between SNPs can account for some of the missing heritability of complex diseases and should be considered when undertaking feature selection. Indeed, studies have demonstrated the benefits to predictive power of ML approaches that consider feature interactions when compared to those that only consider simple additive risk contributions (Couronné et al., 2018; Ooka et al., 2021). However, searching for relevant feature interactions undoubtedly comes with additional computational costs. As such, deciding whether different aspects of it must be done (i.e., searching for relevant interactions) is a problem-specific question that depends upon the nature of the input data and the *a priori* assumptions of the underlying mechanisms of the disease. For example, if the genetic data originates from whole-genome sequencing (WGS), or a genotyping array, and the target phenotype is a complex disease (i.e. best explained by non-linear interactions between loci) then using a feature selection approach that considers interactions will be beneficial. By contrast, if the input genetic data does not uniformly cover the genome (i.e., the density of the SNPs is much higher in known disease associated loci; e.g. Immunochip genotyping array) then interactions may not aid the selection as the lack of data leads to potentially important interactions with SNPs outside known disease associated loci being missed. Furthermore, not all diseases are recognized as involving complex epistatic effects. In such cases, searching for feature interactions might lead to additional computation complexity without obvious predictive benefits. For example, Romagnoni et al. (Romagnoni et al., 2019) reported that searching for possible epistatic interactions did not yield a significant increase in predictive accuracy for Crohn’s disease. Notably, the authors concluded that epistatic effects might make limited contributions to the genetic architecture of Crohn’s disease, and the use of the Immunochip genotyping array might have caused interaction effects with SNPs outside of the known autoimmune risk loci to have been missed.

The goal of feature selection is to select a minimum subset of features (which includes individually relevant and interacting features) that can be used to explain the different classes with as little information loss as possible (Yu and Liu, 2004). It is possible that there are multiple possible minimum feature subsets due to redundancies. Thus, this is “a minimum subset” and not “the minimum set.”

In the remainder of this manuscript we discuss the advantages and disadvantages of representative filter, wrapper, and embedded methods of feature selection (Section 2). We then assess expansions of these feature selection methods (e.g. hybrid, ensemble, and integrative methods; Sections 3.1–3.2) and exhaustive search methods for higher-order (≥3) SNP-SNP interaction/epistasis effects (Section 4).

## 2 Feature Selection Techniques

The feature selection methods that are routinely used in classification can be split into three methodological categories (Guyon et al., 2008; Bolón-Canedo et al., 2013): 1) filters; 2) wrappers; and 3) embedded methods (Table 1). These methods differ in terms of 1) the feature selection aspect being separate or integrated as a part of the learning algorithm; 2) evaluation metrics; 3) computational complexities; 4) the potential to detect redundancies and interactions between features. The particular strengths and weaknesses of each methodological category mean they are more suitable for particular use cases (Saeys et al., 2007; Okser et al., 2013; De et al., 2014; Remeseiro and Bolon-Canedo, 2019) (Table 1).

**TABLE 1:** Strengths, weaknesses, and examples of the three main feature selection categories.

Feature Selection Method	Strengths	Weaknesses	Examples
Filter—Univariate	- Fast and scalable	- Feature dependencies not modeled	- χ^2^/chi-squared test
	- Independent of classifier	- Interaction with classifer not modeled	- Fisher’s exact test
	- Reduce risk of overfitting		- Pearson correlation
			- Information gain
			- *t*-test
			- Mann-Whitney U test
Filter—Multivariate	- Can model feature dependencies	- Slower and not as scalable as univariate filters	- Fast correlation-based filter (FCBF) (Yu and Liu, 2004)
	- Independent of the classifier	- Interaction with classifier not modeled	- Minimal-redundancy-maximal-relevance (mRMR) (Peng et al., 2005)
	- Less risk of overfitting		- Relief-based algorithms (Kira and Rendell, 1992; Kononenko, 1994; Moore and White, 2007; Greene et al., 2009; Greene et al., 2010; Granizo-Mackenzie and Moore, 2013; Urbanowicz et al., 2018a)
Wrapper	- Model feature dependencies	- Slower than filter and embedded methods	- Sequential forward and backward selection (Kittler, 1978)
	- Better performance than filter method	- More prone to overfitting	- Randomized hill climbing (Skalak, 1994)
	-Model interaction with classifier	- Selected features are classifier dependent	- Genetic algorithm (Hayes-Roth, 1975)
			- Recursive feature elimination
Embedded	- Model feature dependencies	- Slower than filter methods	- Random forest (Breiman, 2001)
	- Faster than wrapper method	- Selected features are classifier dependent	- Lasso (L1) or elastic net regression
	- Model interaction with classifier		

### 2.1 Filter Methods for Feature Selection

**FIGURE 5:**
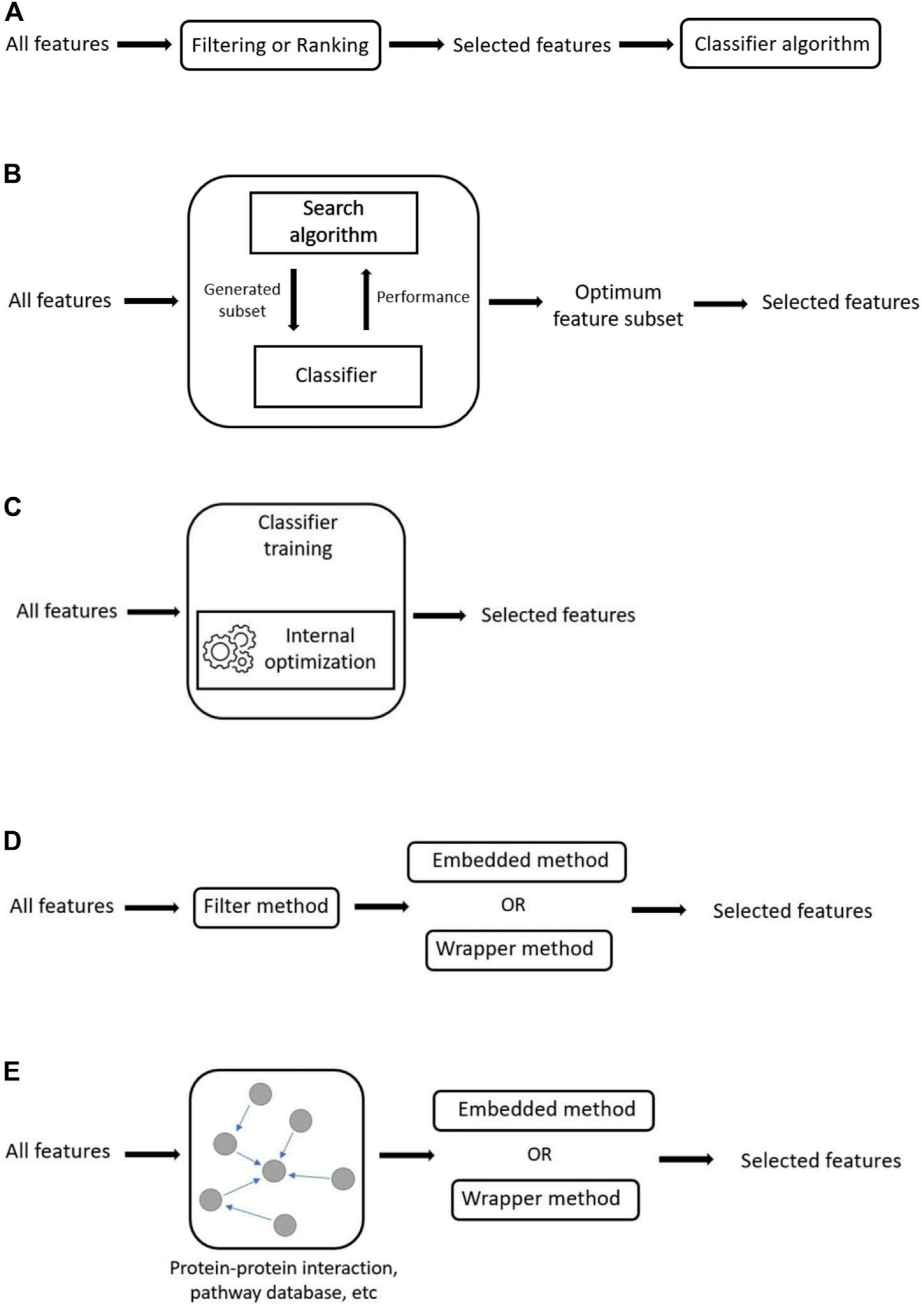
Generalized illustrations of methods. **(A)** Schematic of filter method, where feature selection is independent of the classifier. **(B)**The wrapper method. Feature selection relies on the performance of the classifier algorithm on the various generated feature subsets. **(C)** The embedded method. In embedded methods, feature selection is integrated as a part of the classifier algorithm. **(D)** Hybrid methods. In hybrid methods, features are reduced through the application of a filter method before the reduced feature set is passed through a wrapper or embedded method to obtain the final feature subset. **(E)** Integrative methods. In integrative methods, external information is used as a filter to reduce feature search space before the reduced feature set is passed through a wrapper or embedded method to obtain the final feature subset.

Filter methods use feature ranking as the evaluation metric for feature selection. Generally, features are ranked based on their scores in various statistical tests for their correlation with the class. Features that score below a certain threshold are removed, while features that score above it are selected. Once a subset of features is selected, it can then be presented as an input to the chosen classifier algorithm. Unlike the other feature selection methods (wrapper and embedded), filter methods are independent/separate from the classifier algorithm (Figure 5A). This separation means that filter methods are free from classifier’s bias which reduces overfitting. However, this independence also means that interaction with the classifier is not considered during feature selection (John et al., 1994). Thus, the selected feature set is more general and not fine-tuned to any specific classifier (Zhang et al., 2013). This lack of tuning means that filter methods tend to produce models that have reduced predictive performance compared to those produced by wrapper or embedded methods. The main advantage of filter methods over other feature selection methods is that they are generally less computationally demanding, and thus can easily be scaled to very high dimensional data (e.g. SNP genotype datasets).

Existing filter methods can be broadly categorized as either univariate or multivariate. Univariate methods test each feature individually, while multivariate methods consider a subset of features simultaneously. Due to their speed and simplicity, univariate methods (e.g., χ2 test, Fisher’s exact test, information gain, Euclidean distance, Pearson correlation, Mann-Whitney U test, *t*-test, etc...) have attracted the most attention in fields that work with high dimensional datasets (Saeys et al., 2007; Bolón-Canedo et al., 2014). However, since each feature is considered separately, univariate methods only focus on feature relevance and cannot detect feature redundancy, or interactions. This decreases model predictor performance because: 1) the inclusion of redundant features makes the feature subset larger than necessary; and 2) ignoring feature interactions can lead to the loss of important information.

More advanced multivariate filter techniques, including mutual information feature selection (MIFS) (Battiti, 1994), minimal-redundancy-maximal-relevance (mRMR) (Peng et al., 2005), conditional mutual information maximization (CMIM) (Schlittgen, 2011), and fast correlation-based filter (FCBF), (Yu and Liu, 2004), have been developed to detect relevant features and eliminate redundancies between features without information loss. Other algorithms like BOOST (Wan et al., 2010), FastEpistasis (Schüpbach et al., 2010), and TEAM (Zhang et al., 2010) have been designed to exhaustively search for all possible feature interactions. However, they are restricted to two-way (pairwise) interactions and they cannot eliminate redundancy. More recent algorithms (e.g., the feature selection based on relevance, redundancy and complementarity [FS-RRC] (Li et al., 2020), Conditional Mutual Information-based Feature Selection considering Interaction [CMIFSI] (Liang et al., 2019)) have been demonstrated to be able to detect feature interactions and eliminate redundancies. However, again, they are mostly constrained to pair-wise feature interactions. Another popular family of filter algorithms is the Relief-based algorithm (RBA) family (e.g., Relief (Kira and Rendell, 1992), ReliefF (Kononenko, 1994), TURF (Moore and White, 2007), SURF (Greene et al., 2009), SURF* (Greene et al., 2010), MultiSURF (Urbanowicz et al., 2018a), MultiSURF* (Granizo-Mackenzie and Moore, 2013), etc…). Relief does not exhaustively search for feature interactions. Instead, it scores the importance of a feature according to how well the feature’s value distinguishes samples that are similar to each other (e.g., similar genotype) but belong to different classes (e.g., case and control). Notably, RBAs can detect pair-wise feature interactions, some RBAs (e.g., ReliefF, MultiSURF) can even detect higher order (>2 way) interactions (Urbanowicz et al., 2018a). However, RBAs cannot eliminate redundant features. Different RBAs have been reviewed and compared previously (Urbanowicz et al., 2018a; Urbanowicz et al., 2018b).

Despite its advantages, it should be noted that multivariate methods are more computationally heavy than univariate methods and thus cannot as effectively be scaled to very high dimensional data. Furthermore, multivariate filters still suffer from some of the same limitations as univariate filters due to their independence from the classifier algorithm (i.e., it ignores interaction with the classifier). In this context, wrapper and embedded methods represent an alternative way to perform multivariate feature selection while allowing for interactions with the classifier although again there is a computational cost (see Sections 2.2, 2.3).

#### 2.1.1 The Multiple Comparison Correction Problem and Choosing the Appropriate Filter Threshold

Filter methods often return a ranked list of features rather than an explicit best subset of features (as occurs in wrapper methods). For example, univariate statistical approaches like χ2 test and fisher exact test rank features based on *p* value. Due to the large number of hypothesis tests made, relying on the usual statistical significance threshold of *p* < 0.05 will result in a preponderance of type 1 errors (false positive). As an illustration, if we perform hypothesis tests on 1 million SNPs at a *p* value threshold <0.05, we can expect around 50,000 false positives, which is a considerable number. Therefore, choosing an appropriate threshold for relevant features adds a layer of complexity to predictive modelling when using feature selection methods that return ranked feature lists.

For methods that return a *p* value, the *p* value threshold is commonly adjusted by controlling for FWER (family-wise error rate) or FDR (false discovery rate). FWER is the probability of making at least one type 1 error across all tests performed (i.e., 5% FWER means there is 5% chance of making at least one type 1 error across all hypothesis tests). FWER can be controlled below a certain threshold (most commonly <5%) by applying a Bonferroni correction (Dunn, 1961). The Bonferroni correction works by dividing the desired probability of type 1 error *p* (e.g., *p* < 0.05) by the total number of independent hypotheses tested. This is a relatively conservative test that assumes that all the hypotheses being tested are independent of each other. However, this assumption is likely to be violated in genetic analyses where SNPs that are close to each other in the linear DNA sequence tend to be highly correlated due to LD (Figure 3). Thus, the effective number of independent hypothesis tests is likely to be smaller than the number of SNPs examined. Not taking LD into account will lead to overcorrection for the number of tests performed. For example, the most commonly accepted *p* value threshold used in GWAS (*p* < 5 × 10^−8^) is based on a Bonferroni correction on all independent common SNPs after taking account of the LD structure of the genome (Dudbridge and Gusnanto, 2008; Xu et al., 2014). Despite its widespread use in GWAS, this threshold has been criticized for being too conservative, leading to excessive false negatives (Panagiotou and Ioannidis, 2012). Panagiotou et al. (Panagiotou and Ioannidis, 2012) noted that a considerable number of legitimate and replicable associations can have *p* values just above this threshold; therefore, a possible relaxation of this commonly accepted threshold has been suggested.

Alternatively, one can apply *p* value adjustment to control for FDR instead of FWER. Controlling for FDR is a less stringent metric than controlling for FWER because it is the allowed proportion of false positives among all positive findings (i.e., 5% FDR means that approximately 5% of all positive findings are false). Despite potentially including more false positives in the selected features, FDR has been shown to be more attractive if prediction (rather than inference) is the end goal (Abramovich et al., 2006).

FDR can be controlled by applying the Benjamini-Hochberg (B-H) procedure (Benjamini and Hochberg, 1995). However, like the Bonferroni correction, the B-H procedure assumes independent hypothesis tests. To satisfy this assumption, for example, Brzyski et al. (2017) proposed a strategy that clusters tested SNPs based on LD before applying B–H. Alternatively, there also exist procedures that control FDR without making any assumptions such as the Benjamini-Yekutieli (B-Y) procedure (Benjamini and Yekutieli, 2001). However, the B-Y procedure is more stringent, leading to less power compared to procedures that assume independence like B-H (Farcomeni, 2008).

The question remains, when applying a Bonferroni, B-H or B-Y correction, which FWER/FDR threshold is optimum (e.g., 5, 7, or 10%)? In a ML context, this threshold can be viewed as a hyperparameter. Thus, the optimum threshold that produces the best performance can be approximated by cross-validation as a part of the model selection process (Figure 1A, step 5). The threshold for feature selection methods that do not directly produce a *p* value (e.g., multivariate algorithms like mRMR (Peng et al., 2005)) can also be chosen using cross validation (e.g. by taking the top *n* SNPs as the selected features).

### 2.2 Wrapper Methods for Feature Selection

In contrast to filter methods, wrapper methods use the performance of the chosen classifier algorithm as a metric to aid the selection of the best feature subset (Figure 5B). Thus, wrapper methods identify the best-performing set of features for the chosen classifier algorithm (Guyon and Elisseeff, 2003; Remeseiro and Bolon-Canedo, 2019). This is the main advantage of wrapper methods, and has been shown to result in higher predictive performance than can be obtained with filter methods (Inza et al., 2004; Wah et al., 2018; Ghosh et al., 2020). However, exhaustive searches of the total possible feature combination space are computationally infeasible (Bins and Draper, 2001). Therefore, heuristic search strategies across the space of possible feature subsets must be defined (e.g., randomized (Mao and Yang, 2019), sequential search (Xiong et al., 2001), genetic algorithm (Yang and Honavar, 1998; Li et al., 2004), ant colony optimization (Forsati et al., 2014), etc…) to generate a subset of features. A specific classification algorithm is then trained and evaluated using the generated feature subsets. The classification performances of the generated subsets are compared, and the subset that results in the best performance [typically estimated using AUC (area under the receiver operating characteristic curve)] is chosen as the optimum subset. Practically, any search strategy and classifier algorithm can be combined to produce a wrapper method.

Wrapper methods implicitly take into consideration feature dependencies, including interactions and redundancies, during the selection of the best subset. However, due to the high number of computations required to generate the feature subsets and evaluate them, wrapper methods are computationally heavy (relative to filter and embedded methods) (Chandrashekar and Sahin, 2014). As such, applying wrapper methods to SNP genotype data is usually not favored, due to the very high dimensionality of SNP data sets (Kotzyba - Hibert et al., 1995; Bolón-Canedo et al., 2014).

Wrapper methods are dependent on the classifier used. Therefore, there is no guarantee that the selected features will remain optimum if another classifier is used. In some cases, using classifier performance as a guide for feature selection might produce a feature subset with good accuracy within the training dataset, but poor generalizability to external datasets) (i.e., more prone to overfitting) (Kohavi and John, 1997).

Unlike filter methods which produce a ranked list of features, wrapper methods produce a “best” feature subset as the output. This has both advantages and disadvantages. One advantage of this is that the user does not need to determine the most optimum threshold or number of features selected (because the output is already a feature subset). The disadvantage is that it is not immediately obvious which features are relatively more important within the set. Overall, this means that although wrapper methods can produce better classification performance, they are less useful in exposing the relationship between the features and the class.

### 2.3 Embedded Methods for Feature Selection

In an embedded method, feature selection is integrated or built into the classifier algorithm. During the training step, the classifier adjusts its internal parameters and determines the appropriate weights/importance given for each feature to produce the best classification accuracy. Therefore, the search for the optimum feature subset and model construction in an embedded method is combined in a single step (Guyon and Elisseeff, 2003) (Figure 5C). Some examples of embedded methods include decision tree-based algorithms (e.g., decision tree, random forest, gradient boosting), and feature selection using regularization models (e.g., LASSO or elastic net). Regularization methods usually work with linear classifiers (e.g., SVM, logistic regression) by penalizing/shrinking the coefficient of features that do not contribute to the model in a meaningful way (Okser et al., 2013). It should be noted that like many filter methods, decision tree-based and regularization methods mentioned above also return a ranked list of features. Decision tree-based algorithms rank feature importance based on metrics like the Mean Decrease Impurity (MDI) (Louppe et al., 2013). For regularization methods, the ranking of features is provided by the magnitude of the feature coefficients.

Embedded methods are an intermediate solution between filter and wrapper methods in the sense that the embedded methods combine the qualities of both methods (Guo et al., 2019). Specifically, like filter methods, embedded methods are computationally lighter than wrapper methods (albeit still more demanding than filter methods). This reduced computational load occurs even though the embedded method allows for interactions with the classifier (i.e., it incorporates classifier’s bias into feature selection, which tends to produce better classifier performance) as is done for wrapper methods.

Some embedded methods (i.e…, random forest and other decision tree-based algorithms) do allow for feature interactions. Notably, unlike most multivariate filters, tree-based approaches can consider higher-order interactions (i.e., more than two). Historically, random forest is rarely applied directly to whole-genome datasets due to computational and memory constraints (Szymczak et al., 2009; Schwarz et al., 2010). For example, it has been shown that the original Random Forest algorithm (developed by Breiman and Cutler, 2004) can be applied to analyze no more than 10,000 SNPs (Schwarz et al., 2010). Indeed, many applications of random forest have been focused on low-dimensional dataset. For example, Bureau et al. (Bureau et al., 2005), identified relevant SNPs from a dataset of just 42 SNPs. Lopez et al. (López et al., 2018) implemented a random forest algorithm to identify relevant SNPs from a dataset that contains a total of 101 SNPs that have been previously associated with type 2 diabetes.

Nevertheless, recent advances in computational power, together with optimizations and modifications of the random forest algorithm (e.g., the Random Jungle (Schwarz et al., 2010)) have resulted in efficiency gains that enable it to be applied to whole-genome datasets. However, studies have indicated that the effectiveness of random forest to detect feature interactions declines as the number of features increases, thus limiting the useful application of random forest approaches to highly dimensional datasets (Lunetta et al., 2004; Winham et al., 2012). Furthermore, the ability of standard random forest to detect feature interactions is somewhat dependent on strong individual effects, potentially losing epistatic SNPs with a weak individual effect. Several modified random forest algorithms have been developed to better account for epistatic interactions between SNPs with weak individual effect (e.g., T-tree (Botta et al., 2014), GWGGI (Wei and Lu, 2014)). These modified algorithms are still less sensitive than exhaustive search methods (Section 4).

Unlike some multivariate filters (Section 2.1), random forest does not automatically eliminate redundant features. Indeed, Mariusz Kubus (Kubus, 2019) showed that the presence of redundant features decreases the performance of the random forest algorithm. A potential solution to this problem includes filtering out the redundant features before applying random forest [see hybrid method (Section 3.1)]. Another possible solution might be aggregating the information carried by these redundant features (e.g., using haplotypes instead of SNPs to build the model). Some software packages like T-tree (Botta et al., 2014) have a built-in capability to account for redundancy by transforming the input SNPs into groups of SNPs in high-LD with each other.

In contrast to decision tree-based algorithms, penalized methods (e.g., LASSO) can discard redundant features, but it have no built-in ability to detect feature interactions (Barrera-Gómez et al., 2017). Instead, interaction terms must be explicitly included in the analysis (Signorino and Kirchner, 2018). This is commonly achieved by exhaustively including all (usually pairwise) interaction terms for the features. While this approach can be effective for data with low dimensionality, it can be inaccurate and computationally prohibitive in highly dimensional data settings. Two-stage or hybrid strategies that result in reduced search spaces are potential solutions to this problem (Section 3.1).

### 2.4 Which Feature Selection Method Is Optimal?

The “no free lunch” theorem states that in searching for a solution, no single algorithm can be specialized to be optimal for all problem settings (Wolpert and Macready, 1997). This is true for feature selection methods, each of which has its own strengths and weaknesses (Table 1), relying on different metrics and underlying assumptions. Several studies have compared the predictive performance of the different feature selection methods (Forman, 2003; Bolón-Canedo et al., 2013; Aphinyanaphongs et al., 2014; Wah et al., 2018; Bommert et al., 2020). These comparative studies have resulted in the widely held opinion that there is no such thing as the “best method” that is fit for all problem settings.

Which feature selection method is best is a problem-specific question that depends on the dataset being analyzed and the specific goals that the researcher aims to accomplish. For example, suppose the aim is to identify which features are relatively the most important (which can be useful to help uncover the biological mechanism behind the disease). In that case, filter methods are better because they produce a ranked list of features and are the most computationally efficient. If the dataset contains a relatively low number of features (e.g., tens to hundreds), applying wrapper methods likely results in the best predictive performance. Indeed, in this case, model selection algorithms can be applied to identify which wrapper algorithm is the best. By contrast, for the typical SNP genotype dataset with up to a million features, computational limitations mean that directly applying wrapper or embedded methods might not be computationally practical even though they model feature dependencies and tend to produce better classifier accuracy than filter methods.

New feature selection strategies are emerging that either: 1), use a two-step strategy with a combination of different feature selection methods (hybrid methods); or 2), combine the output of multiple feature selection methods (ensemble methods). These strategies take advantage of the strengths of the different feature selection methods that they include.

## 3 Hybrid Methods—Combining Different Feature Selection Approaches

Hybrid methods combine different feature selection methods in a multi-step process to take advantage of the strengths of the component methods (Figure 5D). For example, univariate filter-wrapper hybrid methods incorporate a univariate filter method as the first step to reduce the initial feature set size, thus limiting the search space and computational load for the subsequent wrapper step. In this instance, the filter method is used because of its simplicity and speed. By contrast, the wrapper method is used because it can model feature dependencies and allow interactions with the classifier, thus producing better performance. Typically, a relaxed scoring threshold is used for the filtering step because the main goal is to prioritize a subset of SNPs for further selection by the wrapper method. For example, when using the univariate χ2 test in the initial feature selection step, instead of the genome-wide significance threshold commonly used in GWAS (*p* > 5 × 10^–8^), one might choose a less stringent threshold (e.g., *p* > 5 × 10^–4^), or adjust by FDR instead. While this might result in more false positives, these can be further eliminated and SNPs with weak individual effects, but strong interacting effects will be able to survive the filtering step and thus can be detected by the wrapper method in the subsequent step. Practically, any filter, wrapper, or embedded method can be combined to create a hybrid method.

In a hybrid method, implementing the filter step reduces the feature search space thus allowing for the subsequent use of computationally expensive wrapper or embedded methods for high-dimensional datasets (which might otherwise be computationally unfeasible). For example, Yoshida and Koike (Yoshida and Koike, 2011) presented a novel embedded method to detect interacting SNPs associated with rheumatoid arthritis called SNPInterForest (a modification of random forest algorithm). To accommodate the computational load of the proposed algorithm, the authors first narrowed the feature size from 500,000 SNPs to 10,000 SNPs using a univariate filter before further selection using SNPInterForest.

Wei et al. (2013) built a Crohn’s disease prediction model that employed a single SNP association test (a univariate filter method), followed by logistic regression with L1 (LASSO) regularization (an embedded method). The first filtering step reduced the original feature size from 178,822 SNPs to 10,000 SNPs for further selection with LASSO. The final predictive model achieved a respectable AUC of 0.86 in the testing set.

There is always a trade-off between computational complexity and performance in feature selection. In this context, hybrid methods can be considered a “middle ground” solution between the simple filter method and the more computationally complex but performant wrapper and embedded methods. Indeed, many examples in the literature have shown that a hybrid method tends to produce better performance than a simple filter while also being less computationally expensive than a purely wrapper method. For example, Alzubi et al. (2017) proposed a feature selection strategy using a hybrid of the CMIM filter and RFE-SVM wrapper method to classify healthy and diseased patients. They used SNP datasets for five conditions (thyroid cancer, autism, colorectal cancer, intellectual disability, and breast cancer). The authors showed that generally, the SNPs selected by the hybrid CMIM + RFE-SVM produce better classification performance than using any single filter method like mRMR (Peng et al., 2005), CMIM (Schlittgen, 2011), FCBF (Yu and Liu, 2004), and ReliefF (Urbanowicz et al., 2018b), thus showing the superiority of the hybrid method.

Ghosh et al. (2020) demonstrated that a hybrid filter-wrapper feature selection technique, based on ant colony optimization, performs better than those based solely on filter techniques. The proposed hybrid method was less computationally complex than those based on the wrapper technique while preserving its relatively higher accuracy than the filter technique. Similarly, Butler-Yeoman et al. (2015) proposed a novel filter-wrapper hybrid feature selection algorithm that was based on particle swarm optimisation (FastPSO and RapidPSO). The authors further showed that the proposed hybrid method performs better than a pure filter algorithm (FilterPSO), while being less computationally complex than a pure wrapper algorithm (WrapperPSO).

Hybrid methods still have limitations despite their advantages when compared to purely filter, embedded, and wrapper methods. For example, relevant interacting SNPs with no significant individual effects (i.e., exclusively epistatic) can potentially be lost during the filtering step. This is because most filter methods cannot model feature-feature interactions. This can be mitigated by using filter algorithms that can model feature interactions (Section 2.1).

### 3.1 Integrative Method—Incorporating External Knowledge to Limit Feature Search Space

Integrative methods incorporate biological knowledge as an *a priori* filter for SNP pre-selection (Figure 5E). This enables the researcher to narrow the search space to “interesting” SNPs that are recognized as being relevant to the phenotype of interest. Limiting the search space means limiting the computational complexity for downstream analysis.

To integrate external knowledge, one can obtain information from public protein-protein interaction databases (e.g., IntAct, ChEMBLOR, BioGRID) or pathway databases (KEGG, Reactome). Software (e.g., INTERSNP (Herold et al., 2009)) has also been developed to help select a combination of “interesting” SNPs based on *a priori* knowledge (e.g., genomic location, pathway information, and statistical evidence). This information enables a reduction in the search space to only those SNPs that are mapped to genes that researchers contend are involved in relevant protein interactions or pathways of interest. For example, Ma et al. (2015) successfully identified SNP-SNP interactions that are associated with high-density lipoprotein cholesterol (HDL-C) levels. The search space was reduced by limiting the search to SNPs that have previously been associated with lipid levels, SNPs mapped to genes in known lipid-related pathways and those that are involved in relevant protein-protein interactions. In other examples, the SNP search space has been limited to SNPs that are located within known risk loci. For example, D’angelo et al. (D’Angelo et al., 2009) identified significant gene-gene interactions that are associated with rheumatoid arthritis (RA) by restricting their search to chromosome 6 (a known as risk locus for RA (Newton et al., 2004)) and using a combined LASSO-PCA approach.

An obvious limitation with these types of integrative approaches is the fact that online databases and our current biological knowledge are incomplete. Therefore, relying on external *a priori* knowledge will hinder the identification of novel variants outside our current biological understanding.

### 3.2 Ensemble Method—Combining the Output of Different Feature Selections

**FIGURE 6:**
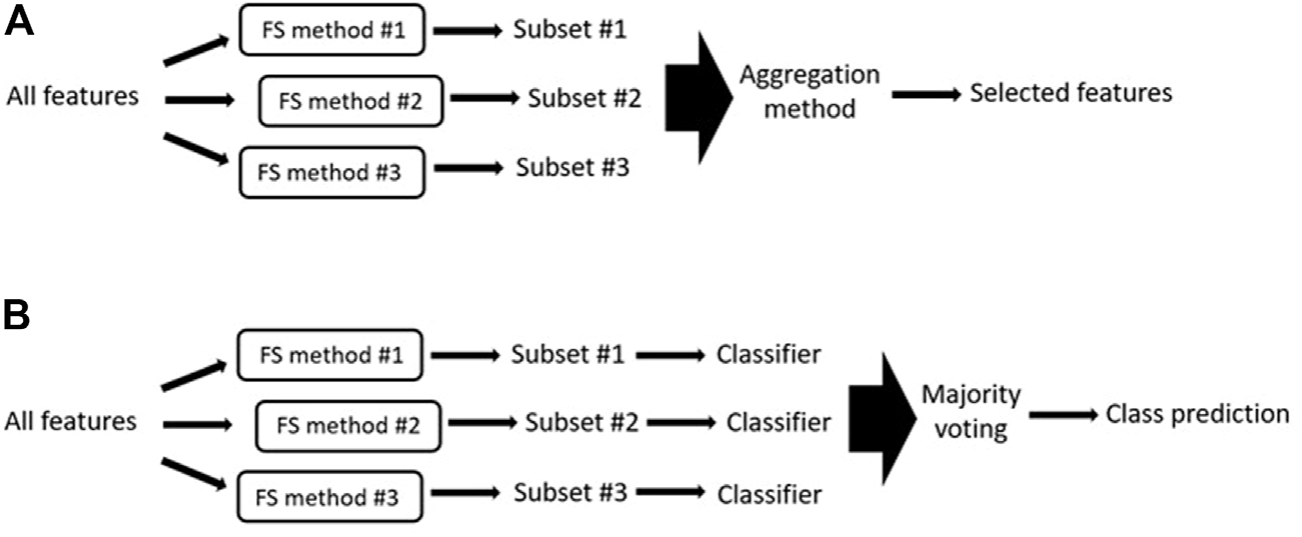
**(A)** Generalized illustration of ensemble methods. In ensemble methods, the outputs of several feature selection methods are aggregated to obtain the final selected features. FS = feature selection. **(B)** Generalized illustration of majority voting system where the different generated feature subsets are used to train and test a specific classifier. The final output is the class predicted by the majority of the classifiers.

Ensemble feature selection methods are based on the assumption that combining the output of multiple algorithms is better than using the output of a single algorithm (Figure 6) (Bolón-Canedo et al., 2014). In theory, an ensemble of multiple feature selection methods allows the user to combine the strengths of the different methods while overcoming their weaknesses (Pes, 2020). This is possible because different feature selection algorithms can retain complementary but different information. Several studies have shown that ensemble feature selection methods tend to produce better classification accuracy than is achieved using single feature selection methods (Seijo-Pardo et al., 2015; Hoque et al., 2017; Wang et al., 2019; Tsai and Sung, 2020). Furthermore, ensemble feature selection can improve the stability of the selected feature set (i.e., it is more robust to small changes in the input data) (Yang and Mao, 2011). Stability and reproducibility of results is important because it increase the confidence of users when inferring knowledge from the selected features (Saeys et al., 2008).

When designing an ensemble approach, the first thing to consider is the choice of individual feature selection algorithms to be included. Using more than one feature selection method will increase the computation time, therefore filter and (to a lesser extent) embedded methods are usually preferred. By contrast, wrappers are generally avoided. Researchers must also make sure that the included algorithms will output diverse feature sets because there is no point in building an ensemble of algorithms that all produce the same results. Several metrics can be used to measure diversity (e.g. pair-wise Q statistics (Kuncheva et al., 2002)).

It is also important to consider how to combine the partial outputs generated by each algorithm into one final output; this is known as the aggregation method. Several aggregation methods have been proposed, the simplest works by taking the union or intersection of the top-ranked outputs of the different algorithms. While taking the intersection seems logical (i.e., if all algorithms select a feature, it might be highly relevant), this approach results in a restrictive set of features and tends to produce worse results than selecting the union (Álvarez-Estévez et al., 2011). To overcome this, other popular aggregation methods assign each feature the mean or median position it has achieved among the outputs of all algorithms and use these positions to produce a final ranked feature subset. The final fusion rank of each feature can also be calculated as a weighted sum of the ranks assigned by the individual algorithms, where the weight of each algorithm is determined based on metrics such as the classification performance of the algorithm (Long et al., 2001). Alternatively, majority voting systems (Bolón-Canedo et al., 2012) (Figure 6B) can be used to determine the final class prediction. In majority voting systems, the different feature subsets generated by each algorithm are used to train and test a specific classifier. The final predicted output is the class that is predicted by the majority of the classifiers (see (Guan et al., 2014; Bolón-Canedo and Alonso-Betanzos, 2019) for reviews about ensemble methods).

Verma et al. (2018) proposed the use of a collective feature selection approach that combined the union of the top-ranked outputs of several feature selection methods (MDR, random forest, MultiSURFNTuRF). They applied this approach to identify SNPs associated with body mass index (BMI) and showed that the ensemble approach could detect epistatic effects that were otherwise missed using any single individual feature selection method.

Bolón-Canedo et al. (2012) applied an ensemble of five filter methods (CFS, Consistency-based, INTERACT, Information Gain and ReliefF) to ten high dimensional microarray datasets. The authors demonstrated that the ensemble of five filter methods achieved the lowest average error for every classifier tested (C4.5, IB1, and naïve Bayes) across all datasets, confirming the advantage of using the ensemble method over individual filters.

## 4 Exhaustive Searches for Higher-Order SNP-SNP Interactions

There are instances where scientists are mainly interested in inference, not prediction (e.g., the research interest lies in interpreting the biology of the selected SNPs). Recently, researchers within the GWAS field have recognized the importance of identifying significant SNP-SNP interactions, especially for complex diseases. The wrapper and embedded methods (e.g., decision tree-based algorithms) that can detect feature interactions (see Section 2.2–2.3) have some limitations: 1). Despite modifications that enable epistasis detection (Section 2.3), random forest-based algorithms are not exhaustive and are still prone to miss epistatic SNPs with low individual effects; 2) wrapper methods return a subset of features but do not identify which are relatively more important than others.

In theory, the most reliable (albeit naïve) way to detect relevant SNP-SNP interactions is by exhaustively testing each possible SNP combination and how it might relate to the phenotype class. Indeed, several exhaustive filter methods have been proposed (see (Cordell, 2009; Niel et al., 2015)). Some examples include, BOolean Operation-based Screening and Testing” (BOOST), FastEpistasis (Schüpbach et al., 2010), and Tree-based Epistasis Association Mapping (TEAM) (Zhang et al., 2010). However, these methods are restricted to testing and identifying pair-wise SNP interactions. Therefore, any epistatic effects of ≥3 orders will be missed. This contrasts with random forest (and many of its modifications), which despite its lower sensitivity (compared to exhaustive filters), can identify higher order interactions.

For higher-order interactions, exhaustive filter methods have been developed (e.g., Multifactor Dimensionality Reduction (MDR) (Ritchie et al., 2001) or the Combinatorial Partitioning Method (CPM) (Nelson et al., 2001)) and shown to be able to detect SNP-SNP interactions across ≥3 orders. However, due to the computational complexity of these analyses, these methods are effectively constrained to a maximum of several hundred features and they cannot be applied to genome-wide datasets (Lou et al., 2007). Goudey et al. (Goudey et al., 2015) estimated that evaluating all three-way interactions in a GWAS dataset of 1.1 Million SNPs could take up to 5 years even on a parallelized computing server with approximately 262,000 cores.

The application of exhaustive methods to genome-wide data can be achieved using an extended hybrid approach (i.e., applying a filter method as a first step, followed by an exhaustive search), or an integrative approach (incorporating external knowledge) that reduces the search space for the exhaustive methods (Pattin and Moore, 2008). For example, Greene et al. (Greene et al., 2009) recommended the use of SURF (a Relief-based filter algorithm) as a filter before using MDR to exhaustively search for relevant SNP interactions. Collins et al. (2013) used MDR to identify significant three-way SNP interactions that are associated with tuberculosis from a dataset of 19 SNPs mapped to candidate tuberculosis genes. Similarly, algorithms that incorporate two-stage strategies to detect high-order interactions have been developed (e.g., dynamic clustering for high-order genome-wide epistatic interactions detecting (DCHE) (Guo et al., 2014) and the epistasis detector based on the clustering of relatively frequent items (EDCF) (Xie et al., 2012)). DCHE and EDCF work by first identifying significant pair-wise interactions and using them as candidates to search for high-order interactions. More recently, swarm intelligence search algorithms have been proposed as an alternative way to look for candidate higher-order feature interactions, prior to application of an exhaustive search strategy. For example, Tuo et al. (2020) proposed the use of multipopulation harmony search algorithm to identify candidate *k*-order SNP interactions to reduce computation load before applying MDR to verify the interactions. Notably, the multi-stage algorithm (MP-HS-DHSI) that Tuo et al. developed is scalable to high-dimensional datasets (>100,000 SNPs), much less computationally demanding than purely exhaustive searches, and is sensitive enough to detect interactions where the individual SNPs have no individual effects (Tuo et al., 2020).

Despite being time demanding, the exhaustive search for pair-wise SNP interaction is possible (Marchini et al., 2005). However, exhaustive searches for higher-order interactions are not yet available. Researchers must resort to hybrid, integrative, or two-stage approaches to reduce the feature space prior to exhaustive search (Table 2). Several (non-exhaustive) embedded methods (e.g., approaches based on decision tree algorithms) have been proposed as viable options to identify SNP interactions and increase the best predictive power of the resulting information. However, the need for an efficient and scalable algorithm to detect SNP-SNP interactions remains, especially for higher-order interactions.

**TABLE 2:** Summary of algorithms reviewed to detect epistasis along with datasets applications, computational time, and memory requirements. Data are taken from three comparative studies, each of which are colour coded differently. N/A, not available.

Method	Algorithm/software	Exhaustive search ?	Detects Higher-order Interaction ?	Dataset	No. SNPs	Time	Mem	References
Filter (multivariate)	BOOST	Yes	No	Colorectal cancer SNPs (CORRECT study)	253,657	5 h	N/A	Kafaie et al. (2021)
	FastEpistasis	Yes	No		253,657	98.5 h	N/A	
	TEAM	Yes	No		253,657	271 h	N/A	
Filter (multivariate)	MDR (pair-wise)	Yes	No	Obesity SNPs (MyCode DiscovEHR study)	100,000	25 h	10 Gb	Verma et al. (2018)
	MultiSURF + TURF	No	Yes		100,000	2.3 h	28 Gb	
Embedded (Decision tree-based)	Random Forest (Ranger R package)	No	Yes		100,000	Not feasible	—	
					500	11.4 min	8 Gb	
	Gradient Boosting	No	Yes		100,000	Not feasible	—	
					500	7.8 min	8 Gb	
Filter (multivariate)	MDR (up to 5 order interactions)	Yes	Yes	WTCCC—T1D	2,184	Not feasible	—	Wei and Lu (2014)
					20	2 min	56 Mb	
	BOOST	Yes	No		2,184	14 s	5 Mb	
Embedded (Decision tree-based)	Random Jungle	No	Yes	WTCCC—T1D	2,184	12 min	110 Mb	
	GWGGI-TAMW	No	Yes	WTCCC—T1D	2,184	3 min	7 Mb	
				WTCCC—CAD	459,000	10 h	738 Mb	
	GWGGI-LRMW	No	Yes	WTCCC—T1D	2,184	1.5 min	7 Mb	
				WTCCC –CAD	459,000	3.5 h	731 Mb	

## 5 Conclusion

Supervised ML algorithms can be applied to genome-wide SNP datasets. However, this is often not ideal because the curse of dimensionality leads to long training times and production of an overfitted predictive model. Therefore, the reduction of the total feature numbers to a more manageable level by selection of the most informative SNPs is essential before training the model.

Currently, no single feature selection method stands above the rest. Each method has its strengths and weaknesses (Table 1, Table 3, discussed in Section 2.4). Indeed, it is becoming rarer for researchers to depend on just a single feature selection method. Therefore, we contend that the use of a two-stage approach or hybrid approach should be considered “best practice.” In a typical hybrid approach, a filter method is used in the first stage to reduce the number of candidate SNPs to a more manageable level, so that more complex and computationally heavy wrapper, embedded, or exhaustive search methods can be applied. Depending on the available resources, the filter used should be multivariate and able to detect feature interactions. Alternatively, biological knowledge can be used as an *a priori* filter for SNP pre-selection. Multiple feature selection methods can also be combined in a parallel scheme (ensemble method). By exploiting strengths of the different methods, ensemble methods allow better accuracy and stability than relying on any single feature selection method.

**TABLE 3:** Advantages, limitations, and references for the feature selection algorithms reviewed in this paper.

Method	Algorithms/softwares	Advantages	Limitaitons	References
Filter (multivariate)	MIFS, mRMR, CMIM, FCBF	- Can remove redundant features	- Ignores feature interaction	Battiti, (1994), Peng et al. (2005), Yu and Liu (2004), Schlittgen, (2011)
		- Can be used for high-dimensional data	- Not exhaustive	
	FS-RRC, CMIFSI	- Can detect pair-wise feature interaction	- Not exhaustive	Liang et al. (2019), Li et al. (2020)
		- Can remove redundant features		
	BOOST, FastEpistasis, TEAM	- Performs exhaustive search	- Cannot remove redundant features	Schüpbach et al. (2010), Wan et al. (2010), Zhang et al. (2010)
		- Can detect pair-wise feature interaction	- Computationally expensive (relative to non-exhaustive filters)	
	Relief-based Algorithms: Relief, ReliefF, TURF, SURF, SURF*, MultiSURF, MultiSURF*	- Can detect pair-wise feature interactions	- Not exhaustive	Kira and Rendell, (1992), Kononenko, (1994), Moore and White, (2007), (Greene et al., 2009), Granizo-Mackenzie and Moore, (2013), Greene et al. (2010), Urbanowicz et al. (2018a)
		- Some algorithms (ReliefF, MultiSURF) can detect higher-order interactions	- Cannot remove redundant features	
	MDR, CPM	- Performs exhaustive search	- Computationally very expensive for higher-order interactions (Cannot be applied to high-dimensional data)	Ritchie et al. (2001), Nelson et al. (2001)
		- Can detect higher-order interactions		
	DCHE, EDCF	- Performs exhaustive search	-Potentially lose feature interactions that do not have significant pair-wise effect	Xie et al. (2012), Guo et al. (2014)
		- Can detect higher-order interactions		
		- Can remove redundant features		
Embedded	Random Jungle, GWGGI	- Can detect higher-order interactions	- Not exhaustive	Schwarz et al. (2010), Wei and Lu, (2014)
		- Feature selection and prediction model are made simultaneously	- Cannot remove redundant features	
	T-Tree	- Can detect higher-order interactions	- Not exhaustive	Botta et al. (2014)
		- Feature selection and prediction model are made simultaneously		
		- Can remove redundant features		
